# Potential Wildlife Sentinels for Monitoring the Endemic Spread of Human Buruli Ulcer in South-East Australia

**DOI:** 10.1371/journal.pntd.0002668

**Published:** 2014-01-30

**Authors:** Connor Carson, Caroline J. Lavender, Kathrine A. Handasyde, Carolyn R. O'Brien, Nick Hewitt, Paul D. R. Johnson, Janet A. M. Fyfe

**Affiliations:** 1 Victorian Infectious Diseases Reference Laboratory (VIDRL), North Melbourne, Victoria, Australia; 2 Department of Zoology, The University of Melbourne, Parkville, Victoria, Australia; 3 Faculty of Veterinary Science, The University of Melbourne, Parkville, Victoria, Australia; 4 Communicable Disease Prevention and Control, Department of Health, Melbourne, Victoria, Australia; 5 Infectious Diseases Department, Austin Health, Heidelberg, Victoria, Australia; Fondation raoul Follereau, France

## Abstract

The last 20 years has seen a significant series of outbreaks of Buruli/Bairnsdale Ulcer (BU), caused by *Mycobacterium ulcerans*, in temperate south-eastern Australia (state of Victoria). Here, the prevailing view of *M. ulcerans* as an aquatic pathogen has been questioned by recent research identifying native wildlife as potential terrestrial reservoirs of infection; specifically, tree-dwelling common ringtail and brushtail possums. In that previous work, sampling of environmental possum faeces detected a high prevalence of *M. ulcerans* DNA in established endemic areas for human BU on the Bellarine Peninsula, compared with non-endemic areas. Here, we report research from an emergent BU focus recently identified on the Mornington Peninsula, confirming associations between human BU and the presence of the aetiological agent in possum faeces, detected by real-time PCR targeting *M. ulcerans* IS*2404*, IS*2606* and KR. *Mycobacterium ulcerans* DNA was detected in 20/216 (9.3%) ground collected ringtail possum faecal samples and 4/6 (66.6%) brushtail possum faecal samples. The distribution of the PCR positive possum faecal samples and human BU cases was highly focal: there was a significant non-random cluster of 16 *M. ulcerans* positive possum faecal sample points detected by spatial scan statistics (*P*<0.0001) within a circle of radius 0.42 km, within which were located the addresses of 6/12 human cases reported from the area to date; moreover, the highest sample PCR signal strength (equivalent to ≥10^6^ organisms per gram of faeces) was found in a sample point located within this cluster radius. Corresponding faecal samples collected from closely adjacent BU-free areas were predominantly negative. Possums may be useful sentinels to predict endemic spread of human BU in Victoria, for public health planning. Further research is needed to establish whether spatial associations represent evidence of direct or indirect transmission between possums and humans, and the mechanism by which this may occur.

## Author Summary

*Mycobacterium ulcerans* causes the disfiguring human skin disease Buruli ulcer (BU). The mechanism of transmission and reservoir for human infection remain unknown. In previous research, we reported the detection of *M. ulcerans* DNA in the faeces of possums (small tree-dwelling marsupials) in an area of South-East Australia (the Bellarine peninsula) where the largest recorded outbreak of human BU has been in progress for the last decade. The current study was carried out in a new outbreak area (the Mornington peninsula), and describes the detection of *M. ulcerans* DNA in possum faeces collected from the ground, in locations which correspond closely with the addresses of human BU cases. The association of new human BU cases with areas where *M. ulcerans* positive possum faeces are found contributes further evidence to the possible role of possums as an environmental reservoir of infection. Possums may be useful sentinel animals to monitor the spread of BU in Australia.

## Introduction

**Figure 1:**
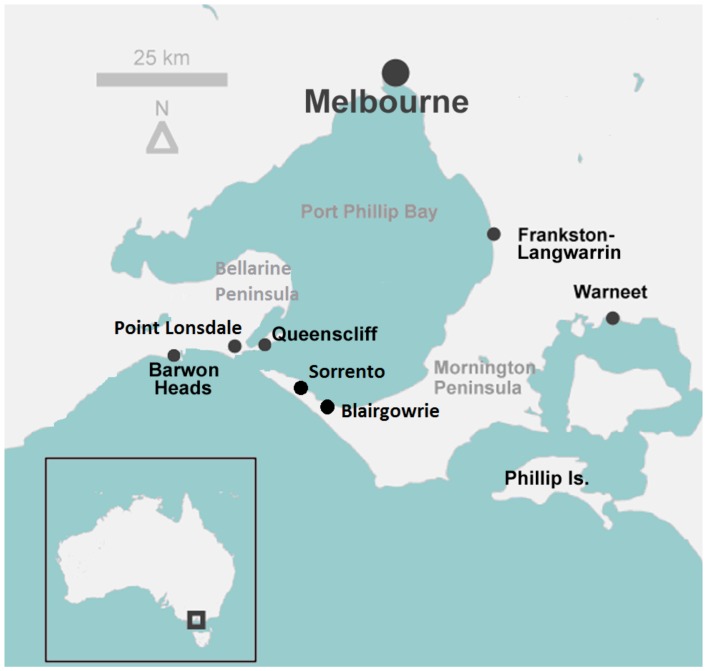
Map showing the Mornington and Bellarine peninsulas, and locations of towns referred to in the text.

*Mycobacterium ulcerans* is an environmental, potentially zoonotic bacterial pathogen, which in humans causes the progressive ulcerative skin condition Buruli Ulcer (BU), a neglected tropical disease which is endemic in at least 30 countries worldwide [1]. The majority of the disease burden is in West and sub Saharan Africa, however there is a significant and ongoing outbreak in temperate south-eastern Australia in the state of Victoria (where the disease is also referred to as Bairnsdale ulcer) [2], [3]. In all settings, the geographic distribution of human BU case clusters is highly focal. The exact method of disease transmission is unknown: BU foci in Africa have been associated with natural bodies of fresh water such as rivers and lakes, prompting the hypothesis that human infection is acquired through skin abrasions by physical contact with contaminated water [4], [5] or from the bites of infected aquatic insects such as water bugs (Naucoridae) [6]. It has also been observed that new endemic areas emerge in areas adjacent to recent soil disturbance and flooding [7]. In south-east Australia, infection is consistently associated with coastal areas [8], [9] and the mechanism of transmission remains elusive, although several studies have indicated that mosquitoes may have a role [2], [10]. The bacterium infects a wide range of terrestrial mammals in Australia, including both domestic animals [11]–[13] and native wildlife [14], [15]. More recently, an extensive survey conducted in an area endemic for human BU on the Bellarine Peninsula (Point Lonsdale; see map, Figure 1) revealed that a large proportion of faecal samples from common ringtail (*Pseudocheirus peregrinus*) and common brushtail (*Trichosurus vulpecula*) possums contained high concentrations of *M. ulcerans* DNA [16]. That study showed a strong association between BU endemicity of an area and the proportion and DNA concentration of *M. ulcerans* positive possum faecal samples. More recently, BU has emerged in a previously non-endemic area of the Mornington Peninsula, in the towns of Sorrento and Blairgowrie (Figure 1) distant from previous historical foci further to the east (Phillip Island, and the Frankston-Langwarrin area of outer Melbourne). From 2006 to the present, 12 new cases of human BU have been confirmed in patients who were either residents (n = 6) or visitors (n = 6) to this region, with no known contact with any of the established endemic areas for BU such as the Bellarine Peninsula, and no recent history of travel to endemic areas either interstate or overseas. In light of previous research showing the potential for possums in BU endemic areas to excrete *M. ulcerans* DNA, the aim of this study was to determine whether the presence and/or relative abundance of *M. ulcerans* DNA in possum faecal samples could be associated with a newly established focus of human BU cases in a previously non-endemic area. We undertook a systematic survey of ground collected possum faeces in the area of the emergent Mornington Peninsula BU focus with the objective to analyze the distribution of *M. ulcerans* positive possum faecal samples and to look for spatial associations with human BU case addresses.

## Materials and Methods

### Study site

The towns of Sorrento (population 1448) and Blairgowrie (population 2161), are located near the western tip of the 750 km^2^ Mornington Peninsula, approximately 90 km south of Melbourne (Figure 1). The terrain is predominantly low-lying coastal scrubland (<50 m above sea level), with an average annual rainfall of approximately 730 mm. There are no substantial water courses or large bodies of fresh water in either of the two towns; open drainage ditches with accumulations of standing water are uncommon. The sample sites in each town consist of similar networks of single-track asphalt or gravel roads with grass verges, connecting rows of large dwellings set in spacious fenced grounds with fairly abundant scrub and tree cover including coastal tea trees (*Leptospermum laevigatum*) as well as numerous introduced cultivar species in gardens. A significant proportion of properties in this region are not occupied by permanent residents, but used as holiday homes or temporary tourist accommodation.

### Human case definition

A case of Buruli Ulcer (BU) was defined as a human patient with at least one suggestive clinical lesion from which *M. ulcerans* DNA was detected by real-time IS*2404* PCR. The identity of the *M. ulcerans* strain was confirmed via Variable Number of Tandem Repeat (VNTR) typing [17] of the cultured isolate or using DNA extracted from the clinical specimen at the Victorian Infectious Diseases Reference Laboratory (VIDRL). A patient was suspected of having acquired BU from the Sorrento/Blairgowrie area if he/she was a resident of (n = 6), or a visitor to (n = 6), that area and had not reported recent contact (<12 months) with any other BU endemic area. Addresses were available for all 6 residents, whereas the addresses of visitors' holiday homes were available in 3 cases. The remaining 3 non-resident patients visited holiday homes of unknown location within the Sorrento/Blairgowrie area.

### Sample collection

Samples of possum faeces were collected from ground level (roadside verges) at points arranged in a predetermined grid pattern 200 m apart. Where there was no tree cover at the indicated grid point (and hence, no possum faeces), samples were collected from the nearest available location where faecal pellets could be found. Faeces originating from common ringtail and common brushtail possums (hereafter referred to as ringtail and brushtail possums) were collected and distinguished based on their characteristic size and shape by experienced field workers and with the aid of a track and scat manual [18]. Where possible intact scats, which had not started to break down due to weather and invertebrates, and estimated to be less than a week old, were selected. The sample spacing interval was chosen in an attempt to minimize resampling of faeces from the same animals between adjacent points, since possums are highly territorial and radio-tracking data show that they generally restrict their movements within a radius of approximately 100 m or less (A. Legione, unpublished data). Faecal samples from each sampling location were stored separately in sterile ziplock plastic bags and transported cool to the laboratory for storage at +4°C prior to DNA extraction, typically within a week of collection.

### DNA extraction and real-time PCR to detect *M. ulcerans*

DNA was extracted from possum faecal material using the FastDNA SPIN Kit for Soil (MP Biomedicals, Solon, OH). Faeces (approximately 100 mg) was added to the kit-supplied Sodium Phosphate and MT Buffer in Lysing Matrix E tubes, and was homogenized for 40 s at setting 6 on a FastPrep Instrument (MP Biomedicals, Solon, OH). Tubes were then centrifuged at maximum speed in a bench microfuge for 10 minutes to pellet debris, before the supernatant was removed and mixed with the supplied protein precipitation solution. After centrifugation at maximum speed for 5 min, 200 µl supernatant was transferred for extraction using an automated robotic system (Corbett X-tractor gene, Qiagen), following the manufacturer's recommendations. Extracted DNA (100 µl) was stored at −20°C. Two microliters of DNA template were used in subsequent real time PCR reactions targeting three independent regions in the *M. ulcerans* genome (IS*2404*, IS*2606* and KR), as described previously [19]. Based on the difference in cycle threshold (Ct) values between IS*2606* and IS*2404* (ΔCt [IS*2606*-IS*2404*]) these assays are able to distinguish between *M. ulcerans* strains, which typically cause disease in mammals, and other members of the *M. ulcerans*/*M. marinum* complex (with fewer copies of IS*2606*) which may be present in the environment, but are not associated with the human outbreak. An estimate of *M. ulcerans* bacterial load per gram of possum faeces was obtained based on the previously established correlations between IS*2404* PCR Ct values and bacterial loads in spiked possum faeces [16]. These calculations enabled comparison of the relative amounts of *M. ulcerans* DNA between samples in the present survey, expressed in 10-fold orders of magnitude up to ≥10^6^ organisms per gram of faeces, and should be considered semi-quantitative rather than absolute. Culture of *M. ulcerans* was not attempted since our previous research has shown this to be an insensitive diagnostic method when applied to possum faeces, due to overgrowth of contaminants [16].

### Variable Number Tandem Repeat (VNTR) typing of *M. ulcerans* DNA in extracts prepared from possum faeces

VNTR typing was performed using 1 µl DNA template in 25 µl reaction volume, using conditions described previously [17]. PCR products were visualized on 2% agarose gel with ethidium bromide staining, and product size was estimated with reference to a 100 bp DNA ladder (Promega, Wisconsin, USA). Products of the expected size were purified using the High Pure PCR Purification Kit (Roche Diagnostics, Australia) and sequenced using the BigDye Terminator v3.1 Cycle Sequencing Kit (Applied Biosystems, Foster City, CA) according to the manufacturer's instructions. VNTR sequences were compared with those from a well characterized Victorian *M. ulcerans* isolate (Strain Ref: MU_JKD8049), which was obtained from a BU patient linked to Point Lonsdale in 2004 [20].

### Statistical analysis

Scan statistics were used to detect and evaluate clusters of positive possum faecal samples in a purely spatial setting, using a Bernoulli model (binary outcome) [21]. This analysis was carried out in SaTScan V8.0 (http://www.satscan.org/).

## Results

**Figure 2:**
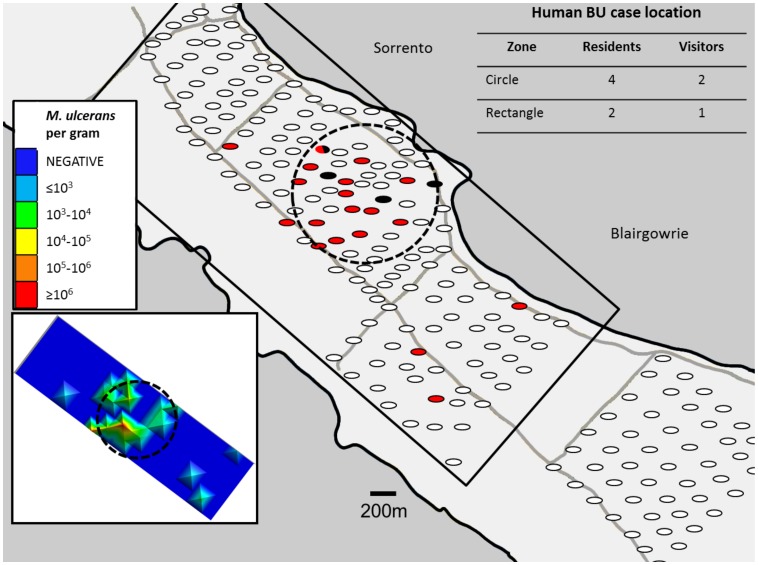
Map showing the distribution of collection sites for possum faeces, indicating the locations of positive samples for *Mycobacterium ulcerans* DNA by real-time IS*2404* PCR. The locations of positive ringtail faecal samples are shown as red ovals; positive brushtail samples are shown as black ovals: negative samples are shown in white. The dotted circle shows a significant non-random clustering of *Mycobacterium ulcerans* positive possum faecal samples identified by spatial scan statistics (*P*<0.0001; 16/30 possum faecal sample positives within a circle of radius 0.42 km). 4/6 residential addresses of human BU cases and 2/3 non-resident addresses fall within the radius of the cluster identified above. Addresses of holiday houses were unavailable for 3 non-resident BU cases. The inset figure (bottom left) depicts a heat map showing possum faecal bacterial loads of samples within the black rectangle, estimated from IS*2404* real-time PCR signal strength, ranging from negative (dark blue) to ≥10^6^ *M. ulcerans* per gram of faeces (red).

*Mycobacterium ulcerans* DNA was detected by real-time PCR in 20/216 (9.3%) ground collected ringtail possum faecal samples and 4/6 (66.6%) brushtail possum faecal samples. There was a significant non-random clustering of positive possum faecal samples identified by spatial scan statistics (*P*<0.0001; 16/30 samples were positive within a circle of radius 0.42 km; see Figure 2). There was a visually apparent spatial correlation between the occurrence of positive possum faeces and the addresses of 6 human BU cases. Four patients who resided locally and two patients who had holiday homes in the area were located within the cluster radius described above: due to requirements of patient confidentiality we are unable to show the specific locations of patients' addresses in Figure 2. Due to the small number of human cases to date, it is not yet possible to confirm statistically significant clustering of human BU cases in this area. Additionally, one human case (resident) was located adjacent to an outlier positive faecal sampling point (again we are unable to depict this case location due to confidentiality requirements) in an area of predominantly negative possum faecal samples. Finally, two human BU case addresses were located in areas where possum faeces was not sampled (one holiday house address, and one resident). The calculated values for Δ*Ct* (IS*2606*-IS*2404*) from *M. ulcerans* PCR positive faecal samples were ≤3.32 (95% CI = 1.56–2.61), confirming that all the sequences detected were attributable to *M. ulcerans* and not another member of the *M. ulcerans/M. marinum* complex which typically give higher Δ*Ct* values (95% CI = 6.94–8.07) [19]. IS*2404* real-time PCR Ct values ranged from 24–39, corresponding with *M. ulcerans* burdens in faeces (estimated as described previously) ranging from ≥10^6^ to 100 organisms per gram of possum faeces. The median estimated bacterial load in *M. ulcerans* positive ringtail faeces was 10^3^–10^4^ organisms per gram, which was similar to the median load in positive brushtail faeces. The two faecal samples with the highest *M. ulcerans* DNA concentrations (≥10^6^ organisms per gram) were from ringtail possums sampled within the cluster of positive possum faecal samples as described above. The DNA concentration in these two samples was sufficiently high to allow sequencing of VNTR locus 14 (both samples) and 9 (1 sample only). The nucleotide sequences obtained were identical to those from the strain of *M. ulcerans* causing human BU disease in Victoria (MU_JKD8049).

## Discussion

Human BU incidence in south-eastern Australia is on the increase, particularly in the last two decades [2], [8]. The progressive extension of the westernmost extremity of the endemic area from the original Bairnsdale region more than 260 km to the east, and the frequent emergence of new endemic foci are current public health concerns. In the most recently studied focus on the Bellarine Peninsula, BU was first reported in 1998, and is now endemic in three small towns near the Eastern tip of the peninsula: in Point Lonsdale (see Figure 1 for location map), the infection rate calculated in 2011 (26 cases) was equivalent to 770/100,000 population (C. Lavender, unpublished data). Tracking of the geographic shift of endemic areas (such as the recent emergence of BU on the western extremity of the Mornington Peninsula) using traditional epidemiological survey methods is complicated by the long incubation period of the disease in humans (median 4.5 months, IQR = 109–160 days) [22], requiring time-consuming analysis of patients' historical movement patterns over an extended period to identify their exposure location. This is particularly challenging in patients who acquire infection from very brief visits to an endemic area as in one documented case, during a stay lasting only a few hours [3]. Further complications in tracking the location of patients' exposure arise because many BU-affected areas are popular holiday resorts that experience high numbers of visiting non-residents particularly in summer. Conversely, survey sampling of roadside-collected possum faeces is straightforward, detection of *M. ulcerans* DNA by real-time PCR can be done within hours using an automated robotic platform, and such a sampling procedure does not raise issues of informed consent, since it does not require examination or interview of human patients. There is increasing interest in the use of wildlife sentinels to monitor the emergence and spread of a number of zoonotic diseases such as West Nile disease, rabies, and anaplasmosis [23], [24]. As a first step towards validating possum faecal surveys as a public health tool to monitor BU emergence, we show here that detection of *M. ulcerans* DNA in possum faeces was associated with a recent outbreak of BU in a previously non-endemic area of the Mornington Peninsula. A significant non-random cluster of *Mycobacterium ulcerans* PCR positive possum faeces was closely adjacent to the addresses of 6 of the total 9 Sorrento/Blairgowrie human BU patients for whom we have obtained residential and holiday home addresses, and the highest *M. ulcerans* bacterial loads in possum faeces coincided with this cluster. It should be noted that in the present study, sampling was carried out on roadside verges underneath overhanging branches of trees growing along the fence line of residences: we cannot rule out the possibility that conditions within fences and boundaries differ from those outside, however this seems unlikely since possum movement is not restricted by such artificial barriers at ground level as being arboreal, they are highly adapted for climbing. Although it was not possible to accurately determine the age of the individual faecal pellets, all were collected from areas exposed to rain and invertebrates which increase the rate of degradation of such samples [25] and on this basis were estimated to be up to a week old. Since pre-outbreak sampling (before 2006) was not done, it is not yet possible to confirm the temporal relationship between possum and human infections. It is noteworthy that we have identified a small number of positive possum scat in a survey of nearby area of the Mornington peninsula (approximately 2 km distant from the present study site) which as yet has no human BU cases (data not shown) – any developments will be reported in future research. Interestingly, the geographic location of the current outbreak area of Sorrento is adjacent to a new housing development, built on the site of a golf course in the mid-1990s. It is highly likely that significant soil disturbance would have taken place at that time.

VNTR typing showed that the *M. ulcerans* in possum faeces on the Mornington Peninsula was indistinguishable from the strain causing human disease in south-east Australia, as was previously demonstrated in possum faeces collected on the Bellarine Peninsula [16]. It is not yet known whether this finding reflects transmission of *M. ulcerans* between possums and humans, or simply a common environmental source of infection. Consistent with previous research [19], we found that sequencing of VNTR loci could be achieved only from IS*2404* positive faecal samples with high *M. ulcerans* bacterial loads (≥10^6^ organisms per gram in the present study). Also in agreement with previous work sampling possum faeces in Victoria [26], no other members of the *M. ulcerans*/*M. marinum* complex were detected in faecal samples collected in Sorrento and Blairgowrie.

Relative to the ubiquitous nature of ringtail possum faeces in the environment, brushtail possum faecal specimens were found rarely (in 6 sampling grid locations only). However the proportion of *M. ulcerans* IS*2404* positive brushtail faecal specimens was higher than that of ringtail samples (66.6%; 4/6 vs 9.3%; 20/216), and it is interesting that positive samples from both species of possum coincided spatially in Sorrento, adjacent to a focus of human BU. Population survey work would be required to determine if ringtail and brushtail possums do indeed coexist in the outbreak area and not elsewhere, which may support the hypothesis of *M. ulcerans* as a cyclozoonosis.

Conversely, in previous work in Point Lonsdale on the Bellarine Peninsula (for map, see Figure 1), the proportion of positive brushtail samples was lower than that of ringtail possum faecal samples (29%; 8/28 vs 43%; 70/164) [16]. The overall number of sample points with brushtail faecal specimens in Point Lonsdale (n = 28) was greater than in the current study in Sorrento/Blairgowrie (n = 6), despite similar sizes of the two sampling areas (approximately 5 km^2^). It is not known if this finding reflects a higher population density of brushtail possums in a well-established BU endemic area, than in the location of a more recent outbreak: confirmation of this would require a survey of the live possum population to estimate overall numbers using established techniques such as spotlighting or trapping [27].

The limited distribution (i.e. highly focal nature) of both human cases and positive possum samples at Sorrento/Blairgowrie contrast the distribution pattern at Point Lonsdale, where human cases and infected possums were more widespread across the whole township [16]. This distribution pattern probably reflects the recent nature of the outbreak in the former, and the considerably longer term presence of the disease agent in populations of both possums and humans in the latter. It will be insightful to reassess the Sorrento/Blairgowrie site again in several years.

As a future research priority, we need information on the degree to which relative population density of ringtail and brushtail possums influences endemicity and/or emergence of BU in humans. Specifically, longitudinal follow-up is needed of human BU disease incidence, possum population dynamics and the prevalence of possum faecal *M. ulcerans* DNA, in the above described emergent endemic area on the Mornington Peninsula. Live trapping of possums has not yet been done in this area to confirm the presence of possums with *M. ulcerans* positive skin lesions, as described in the previous work on the Bellarine Peninsula [16], as distinct from those showing only positive faecal samples. This distinction is important since faecal shedding could occur by simple ingestion of the pathogen e.g. on vegetation, and subsequent excretion, whereas the development of active clinical disease in possums shows the potential for establishment of a long lasting infectious reservoir host. *Mycobacterium ulcerans* in superficial skin lesions would also be available for uptake by biting insects which could potentially act as vectors of BU, as discussed in previous research [2], [3], Overall, a better understanding of spatial and temporal associations between human and possum *M. ulcerans* infection is likely to be the key to elucidation of the transmission mechanism of BU in south-east Australia.
